# Pharmacy professionals’ perceptions of educational supervision in primary care through the lens of Proctor’s model

**DOI:** 10.1186/s12909-023-04398-8

**Published:** 2023-07-12

**Authors:** Michelle Styles, Ellen Schafheutle, Sarah Willis, Matthew Shaw

**Affiliations:** 1 Centre for Pharmacy Postgraduate Education (CPPE), Division of Pharmacy and Optometry, School of Health Sciences, The University of Manchester, Manchester, England; 2 Centre for Pharmacy Workforce Studies, Division of Pharmacy and Optometry, School of Health Sciences, The University of Manchester, Manchester, England; 3 Alliance Manchester Business School, The University of Manchester, Manchester, England

**Keywords:** Primary care education, Postgraduate education, Workplace based learning, Supervision, Educational supervision, Clinical supervision, Proctor’s model

## Abstract

**Background:**

Educational supervision plays a vital role in postgraduate medical education and more recently in pharmacy and advanced clinical practitioner training in England. Proctor’s three-function model of clinical supervision (consisting of formative, restorative, and normative functions) is assumed to apply to educational supervision, but this has not been tested empirically. The aim of this study was to establish perceptions of the purpose of educational supervision from the perspective of primary care pharmacy professionals enrolled on a national training pathway in England.

**Methods:**

Using a mixed methods design, data were collected using a validated 25-item online survey and respondents were invited to add comments explaining their responses. The survey was sent to all 902 learners enrolled on a postgraduate training pathway for pharmacy professionals working in primary care. Principal components analysis (PCA) was used to interpret patterns in the survey data, and framework analysis of qualitative free text comments was used to identify themes and aid interpretation of quantitative findings.

**Results:**

One hundred eighty-seven pharmacy professionals responded (response rate 20.7%). PCA extracted three factors explaining 71.5% of the total variance. Factor 1 corresponded with survey items linked to the formative function of Proctor’s model, while factor 2 corresponded with survey items linked to the restorative function. No items corresponded with the normative function. Framework analysis of qualitative free-text comments identified two themes: learning support, which corresponded with factor 1; and personal support, which corresponded with factor 2.

**Conclusions:**

This study identified that pharmacy professionals perceived educational supervision to perform two functions, formative (educational) and restorative (pastoral), but did not perceive it to perform a normative (surveillance) function. Educational supervision has the potential to support allied health professionals advancing their roles and we suggest the need for more research to develop models of effective educational supervision which can inform practice.

## Background

Educational supervision is an integral part of postgraduate medical specialist training in the United Kingdom (UK) [1] and more recently in advanced clinical practitioner [2] and postgraduate pharmacy training in England [3]. However, it remains one of the least researched aspects of clinical education. Educational supervision focuses on review of educational progress and differs from clinical supervision which involves working alongside the learner to ensure that patients remain safe while learners develop the necessary clinical skills [4]. Although there has been much research on clinical supervision in the healthcare professions [5, 6], there has been little research specifically on educational supervision [7]. Using the exemplar of a national primary care education pathway for pharmacy, the aim of this study was to explore pharmacy professionals’ (pharmacists and pharmacy technicians) understanding of the purpose of educational supervision which may also have relevance for other healthcare professions considering educational supervision as part of support structures to facilitate role advancement.

The National Health Service (NHS) Five Year Forward View envisaged an expanded role for pharmacists in general practice (family medicine) to help address workforce challenges, increase access to care and improve health outcomes [8]. Evidence shows that pharmacists employed in general practice undertake a range of non-traditional tasks, using their expert knowledge of medicines to clinically assess and treat patients [8], reduce healthcare and prescribing costs [9, 10] and undertake clinical interventions [11, 12]. In 2015, NHS England (NHSE) established a pilot to support the development of clinical pharmacists in general practice [13]. Recognising the need to ensure patient safety while pharmacists developed the knowledge and skills needed for these roles, NHSE, at the time via Health Education England (HEE), the organisation which recruits, educates and trains the health workforce across England, commissioned the Centre for Pharmacy Postgraduate Education (CPPE) to develop and deliver a structured 18-month workplace-based education programme for 460 pharmacists enrolled on the pilot. Following pilot evaluation [14], NHSE established the Pharmacy Integration Fund to accelerate integration of pharmacy professionals across a wider range of primary care roles including care homes.

Many of those recruited to primary care lacked the necessary clinical assessment skills for patient-facing roles [15, 16] and little guidance was given to general practices on the role. To address this, the CPPE national primary care education pathway included educational supervision as part of a support structure aimed at ensuring learners acquired the necessary knowledge, skills and competencies required for safe practice [3], identified their learning needs [17] and applied learning in the workplace [18]. Learners received quarterly meetings with an educational supervisor during working hours and participated in small group learning sessions with educational supervisors and peers [3]. Educational supervisors were employed by CPPE and perceived the purpose of their role to include helping learners build confidence and transition from other sectors such as hospital and community pharmacy into primary care [19]. While educational supervision in postgraduate medical education has been shown to support, guide and monitor trainee progress through an extended training programme [1, 6, 7], its role in pharmacy education is less clear.

**Fig. 1:**
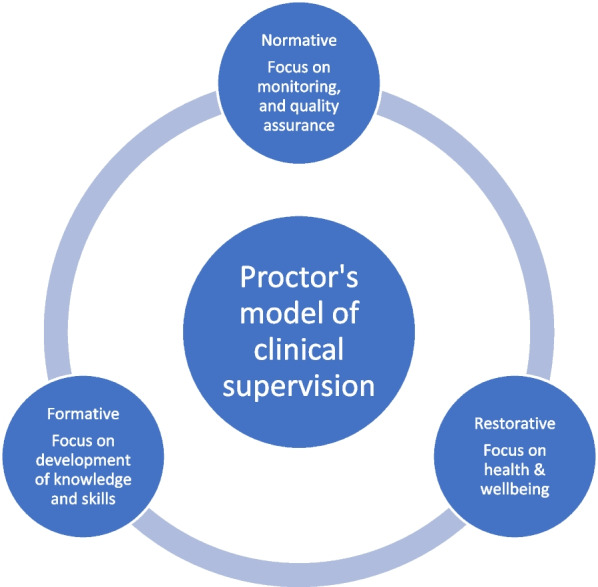
Proctor’s model of clinical supervision

This paper explores perceptions of educational supervision using Proctor’s [20] theoretical model of clinical supervision as its lens. Proctor’s model, based on empirical work by Kadushin [21], describes clinical supervision as having three functions: formative (educational); restorative (pastoral) and normative (administrative and surveillance) (Fig. 1). The formative function links supervision to the development of professional knowledge and skills; the restorative function links it to practitioner wellbeing [22, 23]; and the normative function links it to organisational procedures. The notion of three functions or purposes of clinical supervision is evident across diverse professions including social work [22], educational psychology [24], allied health [25], medicine [6] and nursing [26]. Although there are many models of clinical supervision, particularly in the nursing literature, most are narrative [27] and supervision practices in medicine have little empirical basis [4, 28–30]

The medical education literature assumes that Proctor’s model of clinical supervision also applies to educational supervision but this has not been tested empirically [7]. In the absence of a model specific to educational supervision, Proctor’s model was chosen as the theoretical basis for this study as it is the most widely cited model in the UK healthcare literature [28] and was explicitly referred to in a Department of Health funded evaluation of nursing supervision [29]. Using this model also facilitated comparison of the distinct purposes of clinical and educational supervision.

The purpose of educational supervision has been described as maintenance of training standards [31], provision of feedback [32] and review of educational progress [1] which align with the formative function of Proctor’s model. Educational supervision helps trainees develop self-confidence [31] and provides pastoral support [7, 32], suggesting that it also has a restorative function. However, the purpose of the normative, or surveillance, function in educational supervision is less clear. While quality assurance and trainee performance are important aspects of professional practice, the authors are not aware of any studies having explored whether the normative aspect of Proctor’s model applies to educational supervision. The aim of this study was to establish pharmacy professionals’ perceptions of the purpose of educational supervision and inform development of the theoretical basis for educational supervision.

## Methods

### Design

The study used a mixed methods design with a single data collection phase during which both quantitative and qualitative survey data were collected [33]. Quantitative survey data measured learner ratings of aspects of educational supervision, with qualitative free-text comments invited to provide insights from respondents into the rationale for their ratings. As participants were located throughout England, an online survey allowed data to be collected from a wide range of locations [34].

### Setting and sample

Participants comprised learners enrolled on the CPPE national primary care education pathway for pharmacy professionals. As data collection took place in June 2020 during the COVID-19 pandemic, a census strategy [35] was used, and the survey was sent to all 902 learners enrolled on the pathway. No exclusion criteria were subsequently applied to those meeting initial inclusion criteria.

### Data collection

Data were collected using an online survey [36] hosted on Opinio® which included 25 items related to formative, normative and restorative purposes of supervision. Respondents were asked to rate the extent to which they agreed with each of 25 statements using a five-part Likert scale where 1 was strongly disagree and 5 was strongly agree. Respondents were also invited to explain their ratings with comments.

### Data handling and analysis

Data were downloaded and cleaned and negatively worded items were reverse scored prior to data analysis. Fixed survey items were analysed using SPSS® and total scores calculated. Principal components analysis (PCA), a variant of exploratory factor analysis, was carried out to help interpret patterns in the survey results and provide insight into respondents’ perceptions of the purpose of educational supervision [37].

Responses to open-ended survey items were analysed using framework analysis [38], as it allows both deductive data analysis based on survey questions and inductive analysis of unanticipated themes. Survey question groupings were used for initial deductive coding of comments, for example, three items focused on identifying learning needs and setting objectives, so ‘learning needs’ became a deductive code. Simultaneous inductive coding also took place. After coding 10 cases, 19 deductive codes and 15 inductive codes had been identified. These were reviewed and grouped into thematic categories by looking for repetitions, similarities and differences [39]. The thematic framework was checked by another member of the research team and then used to interpret the output from PCA, thereby integrating qualitative and quantitative data [40].

## Results

One hundred and eighty-seven of the 902 pathway learners who were sent the survey, responded, giving a response rate of 20.7%. Seventy-six respondents (94.1%) rated all 25 items and 134 respondents (71.7%) provided free-text comments. Of the 121 (64.7%) respondents who provided data regarding gender and role, 81.8% (*n* = 99) were female, 84.6% (*n* = 102) were pharmacists and 15.4% (*n* = 19) were pharmacy technicians.

### Factor analysis

PCA extracted three factors with eigenvalues greater than 1. Varimax orthogonal rotation yielded the factor structure shown in Table 1. The rotated solution exhibited a ‘simple structure’ [41] whereby each item had high loadings on one factor only and each factor had high loadings on only some items. The exceptions were items relating to constructive feedback and the importance of making time for educational supervision.

**Table 1:** Orthogonal factor loading matrix for 25 items

	Factor		
	1	2	3
1. ES sessions encourage me to identify my own learning needs	.805	.357	.058
2. ES sessions help me set objectives for my learning & development	.827	.346	.104
3. ES sessions encourage me to manage my own professional development	.821	.358	.021
4. ES sessions help me to develop self-awareness	.780	.302	.192
5. ES sessions help me reflect on my learning, development & progress	.758	.438	.163
6. ES sessions give me time to reflect	.702	.461	.121
7. ES has helped to make me a better practitioner	.790	.330	.147
8. ES sessions challenge me to extend my scope of practice	.787	.290	-.020
9. ES sessions encourage me to apply my learning to practice	.818	.291	.048
10. I receive honest constructive feedback during ES sessions^a^	.574	.540	-.066
11. I receive support and encouragement during ES sessions	.415	.794	.006
12. I feel able to ask for help and support with learning if needed	.475	.688	-.060
13. It is important to establish trust and rapport with my educational supervisor	.188	.607	.087
14. I can discuss work problems that affect my learning during ES sessions	.312	.793	.241
15. I can discuss sensitive issues encountered in my practice during ES sessions	.305	.666	.316
16. I can discuss ideas & concerns about my learning during ES sessions	.402	.796	.115
17. My educational supervisor creates a safe learning environment	.458	.767	.095
18.ES sessions motivate me to progress with my education	.759	.445	.031
19.ES sessions help me identify barriers to learning	.841	.297	-.011
20. ES sessions help me identify solutions to factors hindering learning	.746	.418	-.143
21. ES sessions keep me on track with pathway completion	.424	.700	-.131
22. ES sessions encourage me complete pathway assessments	.391	.738	-.192
23. It is important to make time for ES sessions^a^	.432	.408	.486
24. ES sessions take me away from my real work in practice	-.074	.165	.739
25. ES is unnecessary for experienced practitioners	.112	-.110	.711

^a^ inconsistent loading

### Interpreting factors

Factor 1 comprised survey items linked to the formative function of Proctor’s model, while factor 2 comprised survey items linked to the restorative function. The third factor comprised two items exploring time spent on educational supervision. No items corresponded with the normative function. Factor 2 items were scored higher than factors 1 and 3 (Table 2), suggesting that learners perceived the restorative function to be the most important purpose of educational supervision.

**Table 2:** Learner scores for each factor

	Range of scores	Median score	Mean score	SD	95% confidence intervals	Median score as a percentage of maximum score
**Factor 1**						
Learners *n* = 179^a^	13–65	52	50.2	12.36	47.9–52.4	80
**Factor 2**						
Learners *n* = 176^a^	9–45	41	38.1	7.65		91.1
					36.7–39.5	
**Factor 3**						
Learners *n* = 178^a^	3–15	12	11.79	2.46	11.4–12.2	80

^a^Number of respondents rating items related to this factor

**Fig. 2:**
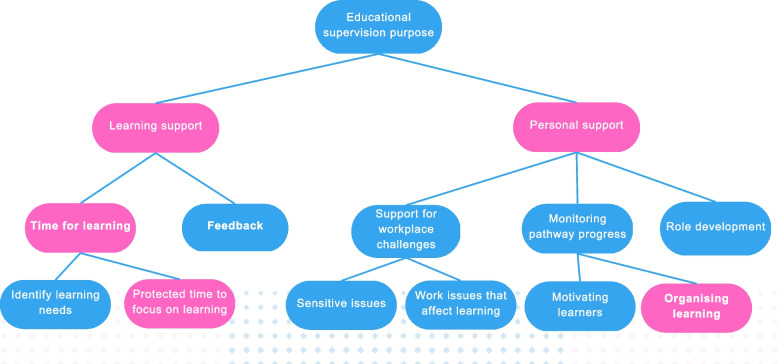
Thematic map for learner perceptions of the purpose of educational supervision. Blue boxes indicate deductive themes and pink boxes indicate inductive themes

Quantitative and qualitative data were integrated in data analysis by using framework analysis of responses to open-ended survey items [40] to support interpretation of the two main factors. Two overarching thematic categories were identified: learning support which corresponds with the formative function and personal support which corresponds with the restorative function (Fig. 2).

Within the theme of learning support, two sub-themes were identified: time for learning; and feedback. Many learners described the difficulties of studying alongside full-time employment but recognised that educational supervision afforded them time away from clinical work to “*reflect and focus*” [Learner 10] on learning. Learners commented that feedback from supervisors and peers helped them develop insight and self-awareness as there was little time for this in the workplace. Although CPPE educational supervisors do not assess clinical performance, some learners suggested they would like to receive more constructive feedback, for example,

*“I feel I need more critical feedback to help me improve.”* [Learner 67].

Within the theme of personal support, three sub-themes were identified – support with workplace challenges, role development, and monitoring pathway progress. Some learners reported that employers had “*unrealistic expectations*” [Learner 9] of what they could do when newly employed in primary care. Several learners described how changes to primary care working practices caused by the COVID-19 pandemic had resulted in additional workplace challenges and commented that educational supervision had helped address these. As educational supervisors were employed directly by CPPE not by general practices or care homes, they were able to act as allies for the learner and were perceived to be impartial, for example,

*“Things you feel you cannot say to a clinical supervisor or practice manager can be voiced to an education supervisor as they are … neutral”.* [Learner 55]

Learners described how educational supervisors had supported them to take on more complex, patient-facing tasks such as medication reviews. One learner commented,

*“It is very easy just to continue doing what you've always done. But discussing with ES makes me think where I could expand my competencies”.* [Learner 20]

Many learners commented that educational supervision sessions had provided opportunities for them to learn about the work of peers in primary care and this had motivated learners to develop their own roles and adopt innovative practices shared by others.

Most learners were positive about the role of educational supervision in monitoring pathway progress. Learners recognised that supervision provided a structure and regular opportunities to review progress, for example,

*“The sessions [with my education supervisor] formalise my learning and also make me realise how much I have actually learnt, which I hadn't fully appreciated until I was made to think about it.”* [Learner 76]

Learners acknowledged the role of the educational supervisor in keeping them on track and described the benefits of having regular reminders to help them organise their learning, for example,

“*I have found it very useful to have an update with my educational supervisor to check that I am on track and progressing with my learning.”* [Learner 31]

The COVID-19 pandemic had disrupted the planned sequence and timing of the pathway for learners and most described feeling reassured by supervisors. Learners were grateful for the practical help provided to summarise achievements and reconfigure plans for pathway completion.

However, there was some contention about who was responsible for ensuring that learners made progress. Many learners felt responsible for their own learning, for example,

“*I didn't feel that it was the education supervisor’s role to keep me on track with pathway. As an adult learner I took that as my responsibility.”* [Learner 48]

These learners resented constant reminders about assessments and pathway completion and did not feel that they needed supervision to motivate them to progress.

## Discussion

Using quantitative and qualitative survey data, this is the first study to explore the relevance of Proctor’s three-function model of clinical supervision to educational supervision. Factor analysis of fixed item responses and subsequent interpretation of these using analysis of free text comments showed that pharmacy professionals perceived educational supervision to perform two functions, formative and restorative, but did not perceive it to perform a normative function, despite the inclusion of survey items specifically relating to monitoring and surveillance such as monitoring pathway progress.

### Formative function

In nursing and allied health, finding time for clinical supervision has been identified as a barrier to effectiveness [42, 43]. Supervision is often not seen as a priority and both learners and supervisors lack clarity on its purpose [5]. Learners enrolled on the primary care pharmacy education pathway are given protected time away from clinical duties for educational supervision and responses to open-ended questions indicated that the provision of ‘learning spaces’ [44] to focus on learning, reflect on and critique practice, and discuss challenging issues away from the workplace was perceived to be an important purpose of educational supervision. Indeed, CPPE educational supervisors had dedicated time to focus on supporting learners.

In medical education, the opportunity to receive feedback is perceived to be a useful formative function of educational supervision [45] and pharmacy professionals in this study recognised its value in helping them improve. Although some learners wanted constructive feedback on clinical performance, this is provided by clinical supervisors, rather than by CPPE educational supervisors, which may explain the inconsistent loading for item 10 relating to receiving constructive feedback from supervisors (Table 1). However, supervisor feedback on educational progress may develop learners’ ability to identify their own learning needs and may be particularly important for pharmacy professionals transitioning to primary care who require support to build confidence in clinical skills, apply learning to practice and take on unfamiliar roles [18].

### Restorative function

The highest score awarded by learners in this study to factor 2, the restorative function, suggests that this was perceived to be the most important purpose of educational supervision, a finding consistent with studies of trainee doctors [7, 46].

Educational supervision reduces workplace stress [31, 47] and as this study took place during the first wave of the COVID-19 pandemic, it is likely that the requirement to set aside time for educational supervision provided respite from clinical work. Workplace stress may also have arisen due to lack of national guidance on the role of pharmacy professionals in primary care [48]. Learners described feeling stressed due to employer pressure to undertake tasks that they did not feel competent to perform. Educational supervision helped to reduce stress by providing learners with opportunities to recognise their limitations and discuss coping strategies. Learners transitioning to primary care roles may be in a ‘liminal space’ [49, 50] as they acquire new knowledge and skills. This can take time and may be an unsettling experience [51] and it is likely that educational supervision supported learners during this transition.

### Normative function

An interesting finding from this study was that although items relating to the normative function of educational supervision such as monitoring educational progress were included in the survey, learners perceived that this supported their learning and did not perceive it as being held to account. Learners took responsibility for their own educational progress and perceived educational supervision to have a supportive function. This could be because educational supervisors for the primary care education pathway are employed by CPPE, not by the learner’s workplace and do not line manage learners, assess performance or oversee clinical work. Studies in medicine [52, 53] and pharmacy [54, 55] have suggested a conflict between the summative nature of formal assessments and the supportive nature of formative interventions such as appraisals. The need to appear competent during assessment can hinder opportunities to learn [56] and it has been suggested that assessments and appraisals should be separate processes [57]. While clinical supervision requires a normative function to ensure patient safety during healthcare professionals’ training, educational supervision appears to have a more nurturing function which supports learners to progress [52–55, 57].

The inclusion of the normative function within Proctor’s model of clinical supervision may be relevant for the medical and nursing professions because in some settings, clinical supervision is provided by line managers. However, this can present conflicts of interest between individual learner needs and responsibility to the organisation and to patients [5]. In contrast, educational supervisors employed by CPPE have no managerial responsibility, allowing learners to focus on identifying and addressing learning gaps without fear of punitive action.

Much of the research on supervision focuses on clinical rather than educational supervision. The terms are often used interchangeably in the literature [58] and it has even been suggested that they should be synonymous [59]. However, this is problematic as it assumes that supervision is a homogenous process. This study suggests that clinical and educational supervision have different purposes, with educational supervision focusing on the formative and restorative functions needed to encourage learners to be open about development needs and discuss strategies to improve.

This study explored whether Proctor’s three-function model of clinical supervision applied to educational supervision in primary care pharmacy education. Clinical supervision’s primary function is formative, but it may also have a normative function in monitoring, standard-setting and assuring patient safety [60–62]. Educational supervision has formative and restorative functions [6, 32] but this study suggests that it does not have a normative (surveillance) function in healthcare professions education.

## Limitations

A limitation of this study is the relatively low response rate. The study took place during the COVID-19 pandemic when learning and supervision were disrupted, and this may have influenced comments. Although pharmacy technicians completed the survey, none provided comments to open-ended survey items.

## Conclusion

It is widely assumed in the medical education literature that Proctor’s three-function model of clinical supervision also applies to educational supervision [6, 63]. However, this study found that educational supervision provided as part of the primary care pharmacy education pathway comprised only two of Proctor’s three functions, formative (learning support) and restorative (personal support) which then suggests that clinical and educational supervision have two distinct purposes. Educational supervision continues to be an under-researched aspect of medical education and findings from this study suggest the need for a programme of research to develop models of effective educational supervision which can inform practice in other pharmacy contexts such as foundation and newly qualified pharmacist training and in other healthcare professions considering using it as part of support structures to facilitate role advancement.

## Data Availability

The dataset generated and analysed during the current study are available in the University College London research data repository available at: https://rdr.ucl.ac.uk/articles/dataset/Education_supervision_in_postgraduate_pharmacy_learner_data/20954347.

## Abbreviations

CPPE: Centre for Pharmacy Postgraduate Education
ES: Educational supervision
HEE: Health Education England
NHSE: NHS England
